# An extended Hilbert transform method for reconstructing the phase from an oscillatory signal

**DOI:** 10.1038/s41598-023-30405-5

**Published:** 2023-03-02

**Authors:** Akari Matsuki, Hiroshi Kori, Ryota Kobayashi

**Affiliations:** 1 Graduate School of Information Science and Technology, The University of Tokyo, Tokyo, 113-8656 Japan; 2 Graduate School of Frontier Sciences, The University of Tokyo, Chiba, 277-8561 Japan; 3 Mathematics and Informatics Center, The University of Tokyo, Tokyo, 113-8656 Japan; 4 JST PRESTO, Saitama, 332-0012 Japan

**Keywords:** Complex networks, Nonlinear phenomena

## Abstract

Rhythmic activity is ubiquitous in biological systems from the cellular to organism level. Reconstructing the instantaneous phase is the first step in analyzing the essential mechanism leading to a synchronization state from the observed signals. A popular method of phase reconstruction is based on the Hilbert transform, which can only reconstruct the interpretable phase from a limited class of signals, e.g., narrow band signals. To address this issue, we propose an extended Hilbert transform method that accurately reconstructs the phase from various oscillatory signals. The proposed method is developed by analyzing the reconstruction error of the Hilbert transform method with the aid of Bedrosian’s theorem. We validate the proposed method using synthetic data and show its systematically improved performance compared with the conventional Hilbert transform method with respect to accurately reconstructing the phase. Finally, we demonstrate that the proposed method is potentially useful for detecting the phase shift in an observed signal. The proposed method is expected to facilitate the study of synchronization phenomena from experimental data.

## Introduction

Rhythmic activity is ubiquitous in biological systems, including cortical networks in the brain^1,2^, human heart and respiratory system^3–5^, circadian rhythm^6,7^, gene expression^8^, and animal gait^9–11^. The phase description approach^12,13^ describes the state of a multi-dimensional nonlinear oscillator using a variable called the phase and derives a reduced phase equation from a nonlinear dynamical system. This approach has promoted the understanding of how a population of nonlinear oscillatory elements can synchronize or form a cluster state. Theoretical studies based on the phase equation have been used to investigate the potential mechanisms underlying synchronization phenomena, including mutual coupling among the elements and the common inputs to the elements^14,15^.

Fundamental questions in complex systems include how a system in the real-world achieves synchronization and what is the essential mechanism that leads to a synchronization state^16^. While the theoretical studies provide potential explanations for the synchronization phenomena, they cannot directly answer these questions. It is essential to reconstruct the instantaneous phase from observed data (e.g., signals or time series) and to infer the phase equation from the reconstructed phase. Many studies have focused on the latter step, that is, they have developed the inference methods for the phase response curve^17–21^ and the coupling function^22–30^ from the phase (various reviews discuss this topic^31,32^). Conversely, a few studies^33,34^ have focused on the former step, i.e., the reconstruction of the instantaneous phase from an observed signal. An accurate phase reconstruction is necessary to study the synchronization phenomena in data because these inference methods assume the perfect phase reconstruction.

There are two primary approaches to reconstructing the instantaneous phase from an oscillatory signal. One simple approach to reconstructing the phase is to use linear interpolation between the subsequent marker events. For example, the phase is defined as 0 or $2\pi$ at the time of the action potential (spike) for neuronal oscillators^17–19^ or a heartbeat^3^. This method can accurately reconstruct the phase when the noise level is not very high. However, this method is not applicable to signals without identifiable marker events, such as neuronal spikes. An alternative phase reconstruction approach is to apply the Hilbert transform to the observed signal^16,35,36^. An advantage of the Hilbert transform method is that it is applicable even when there is no well-defined marker. Consequently, the Hilbert transform method has been applied to a variety of systems, e.g., the respiratory system in human^3,5^, the gene expression in a cell^8^, and the human brain activity^29,37–39^. The limitation of the Hilbert transform method is that it can reconstruct the physically interpretable phase from a limited class of signals, i.e., the narrow band signals^37,40^. Therefore, it is necessary to carefully develop a pre-processing procedure via trial and error, which hinders the application of this method to oscillatory signals. Theoretical studies in signal processing have clarified the mathematical conditions of the signals on which the Hilbert transform method can reconstruct a meaningful phase^37,40,41^. However, only a few attempts have been made to develop a method for reconstructing the phase from more general signals.

**Figure 1:**
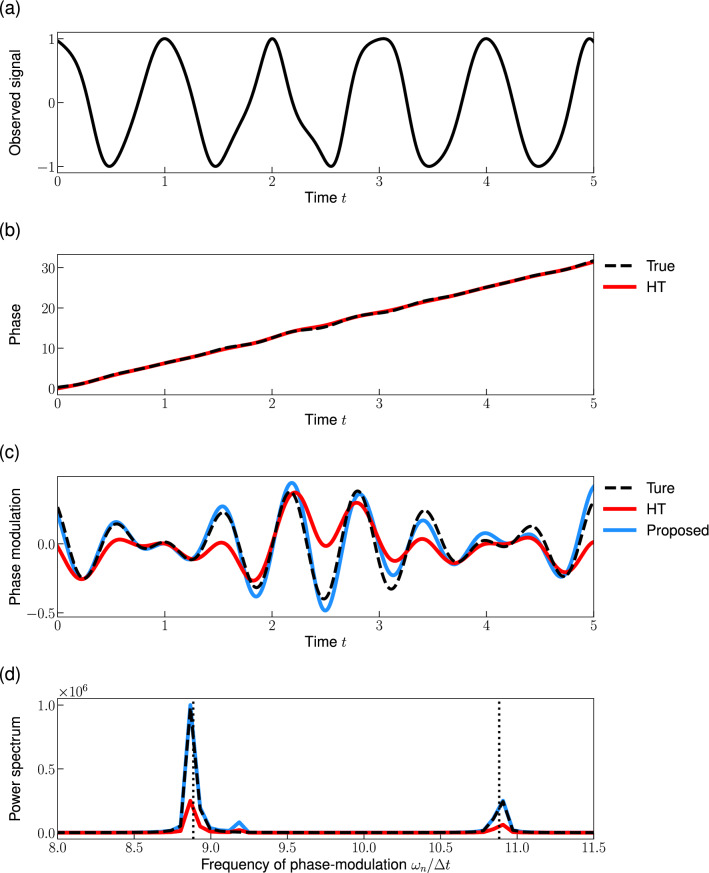
Reconstruction of the instantaneous phase from an observed signal. (**a**) Observed signal *x*(*t*) (Eq. 6) with $u(t) = 0.2 \left( \sin \sqrt{2} \hat{\omega } t + \cos \sqrt{3} \hat{\omega } t \right)$. (**b**) Instantaneous phase $\phi (t)$ (Eq. 6). (**c**) Phase-modulation *u*(*t*). (**d**) Power spectrum of the phase-modulation. The dashed line in (**b**–**d**) represents the true phase, the phase-modulation, and its power spectrum, respectively. The red and blue lines represent the reconstructions by the conventional HT method and the proposed method, respectively. Dotted vertical lines in (**d**) represent the dominant frequencies of the true phase-modulation: $\sqrt{2} \hat{\omega }$ and $\sqrt{3} \hat{\omega }$, where $\hat{\omega }= 2\pi$ is the effective frequency. Note that we plotted a part of the signal and the start time of the plot is redefined as 0.

In this study, we propose an extension of the Hilbert transform method that can reconstruct the interpretable phase from a wider variety of signals. Here, we consider a particular class of signals, called “weakly phase-modulated signals”, which are an extension of the sinusoidal signals from which the conventional Hilbert transform method can reconstruct the phase. These signals are also regarded as a subclass of phase-modulated signals^33,34^. We first demonstrate that this conventional method cannot accurately extract the phase from these signals (Fig. 1). Then, we derive a new algorithm to reconstruct the phase from the phase-modulated signals and empirically show that the proposed method improves the reconstruction performance.

This paper is organized as follows. We first review the conventional Hilbert transform method for reconstructing the instantaneous phase from data. In addition, we illustrate the limitation of the conventional method using an example. Second, we present the proposed method for reconstructing the instantaneous phase and examine the computational complexity of the algorithm. Third, we evaluate the performance of the phase reconstruction and compare its performance with that of the conventional method. Finally, we conclude this study and discuss future directions.

## Results

### Estimating the instantaneous phase from an oscillatory signal

A standard method for reconstructing the instantaneous phase from an oscillatory signal is based on the Hilbert Transform (HT)^16,35,36^. This method calculates the phase from the analytic signal, defined as

1 $$\begin{aligned} \zeta (t) = x(t) + iH\left[ {x(t)}\right] , \end{aligned}$$

where *x*(*t*) and $H\left[ {x(t)}\right]$ are the observed signal and its HT

2 $$\begin{aligned} H\left[ {x(t)}\right] = \pi ^{-1} \mathrm{P.V.}\int _{-\infty }^{\infty } \frac{x(\tau )}{t-\tau } d\tau , \end{aligned}$$

where $\mathrm{P.V.}$ refers to the Cauchy principal value. The HT method reconstructs the instantaneous phase by the argument of the analytic signal

3 $$\begin{aligned} \phi ^{\textrm{H}}(t) = \arg \left[ \zeta (t) \right] . \end{aligned}$$

It is well-known that the HT method can reconstruct the interpretable phase from a particular class of signals. Let us consider the sinusoidal signal

4 $$\begin{aligned} x(t)= A_0 \cos \left( \hat{\omega } t + \phi _0 \right) , \end{aligned}$$

where $\hat{\omega }$ is the effective frequency, and $\phi _0$ is the initial phase. The HT method can perfectly reconstruct the interpretable phase from the signal: $\phi ^{\textrm{H}}(t)= \hat{\omega } t + \phi _0$. Furthermore, it is possible to extend this result to signals with slow amplitude modulation

5 $$\begin{aligned} x(t)= A_L(t) \cos \left( \hat{\omega } t + \phi _0 \right) , \end{aligned}$$

where the amplitude $A_L(t)$ is the low-pass-filtered signal whose Fourier coefficients of the frequency higher than the effective frequency ($f> \hat{\omega }$) vanish. It can be shown^42^ that the HT method can perfectly reconstruct the phase: $\phi ^{\textrm{H}}(t)= \hat{\omega } t + \phi _0$. However, the HT method can only reconstruct the interpretable phase from a particular class of signals, i.e., the narrow band signals^37,40^.

In this study, we extend the HT method for a general type of signal, which we call “weakly phase-modulated signals”

6 $$\begin{aligned} x(t) = A_0 \cos \phi (t), \end{aligned}$$

where $\phi (t)= \hat{\omega } t + u(t)$ is the phase of the signal and *u*(*t*) is a small phase-modulation.

We applied the HT method to a phase-modulated signal (Fig. 1a). Figure 1b demonstrates that the HT method can accurately track the linear trend $\hat{\omega } t$ and estimate the effective frequency $\hat{\omega }$ even from a phase-modulated signal. Note that this method (Fig. 1c, red) cannot accurately reconstruct the phase-modulation $\phi (t)- \hat{\omega } t$. Then, we analyzed the power spectrum of the phase-modulation $\phi (t)- \hat{\omega } t$ to investigate the effect of the HT method. Figure 1d compares the power spectrum of the phase-modulation reconstructed using the HT method with that of the true phase-modulation. We plotted the frequency range of $8.0< f < 11.5$ because the phase-modulation is given by the sum of two sinusoidal functions whose frequencies are $\sqrt{2} \hat{\omega } \approx 8.89$ and $\sqrt{3} \hat{\omega } \approx 10.9$, respectively. The result indicates that the HT method behaves like a low-pass filter, that is, it suppresses the spectral density of the peak frequencies ($f= \sqrt{2} \hat{\omega }, \sqrt{3} \hat{\omega }$). Motivated by this observation, we investigate how the HT method changes the power spectrum in the following subsection. We extend the HT method for reconstructing the instantaneous phase from an oscillatory signal by preserving the power spectrum of the phase-modulation *u*(*t*).

Note that it is critical to reconstruct the phase-modulation *u*(*t*) accurately to study the synchronization mechanism^16^, even though the modulation is small (Fig. 1b,c). Many methods for inferring the phase coupling function rely on the assumption that the phase has been perfectly reconstructed; consequently, the bias in the phase reconstruction may induce serious effects on the inference results.

### Proposed method

As we observed in Fig. 1, the conventional HT method cannot reconstruct an interpretable phase from phase-modulated signals. In this subsection, we extend the HT method to include the phase-modulated signals (Eq. 6).

Let us assume that the signal is sampled at *N* time steps with a constant interval $\Delta t$. We consider a phase modulated signal (Eq. 6) sampled at time $t= k\Delta t$

7 $$\begin{aligned} x[k]:= x(k\Delta t)= A_0 \cos \left( \phi [k] \right) , \end{aligned}$$

where $A_0$ is the amplitude and $\phi [k]:= \phi (k\Delta t)$ is the instantaneous phase at time $t= k\Delta t$.

We can analyze the effect of the phase-modulation on the phase reconstructed via the HT method with the aid of Bedrosian’s theorem. The true phase-modulation $u[k]:= u(k\Delta t)$ and its reconstruction via the HT method $u^{\textrm{H}}[k]:= \phi ^{\textrm{H}}(k\Delta t) - \hat{\omega } k\Delta t$ can be represented as Fourier series:

8 $$\begin{aligned} u[k] = \sum _{n=0}^{N-1} c_n e^{i k \omega _n}, \quad u^{\textrm{H}}[k] = \sum _{n=0}^{N-1} c^{\textrm{H}}_n e^{i k \omega _n}, \end{aligned}$$

where $\omega _n= 2n \pi /N$, and $c_n$ and $c^{\textrm{H}}_n$ are given by the discrete Fourier transform of *u*[*k*] and $u^{\textrm{H}}[k]$, respectively. Assuming that the phase-modulation is small: $\varepsilon := \max _k |u[k]| \ll 1$ and the sampling interval $\Delta t$ is small enough, we can derive a formula that clarifies the relation between the Fourier coefficients ($c_n$ and $c^{\textrm{H}}_n$) by neglecting higher order terms $O(\epsilon ^2)$ (see “Method” for the derivation),

9 $$\begin{aligned} c^{\textrm{H}}_{n} \approx {\left\{ \begin{array}{ll} c_n - \frac{1}{2} \bar{c}_{2m-n} - \frac{1}{2} c_{n+2m} &{} \textrm{for} \quad 0 \le n \le m-1, \\ \frac{3}{4} c_n - \frac{1}{4} \bar{c}_{n} - \frac{1}{2} c_{3n} &{} \textrm{for} \quad n= m, \\ \frac{1}{2} c_n - \frac{1}{2} c_{n+ 2m} &{} \textrm{for} \quad m+1 \le n \le N/2- 2m, \\ \frac{1}{2} c_n &{} \textrm{for} \quad N/2- 2m + 1 \le n \le N/2, \\ \end{array}\right. } \end{aligned}$$

where *m* is an effective frequency index, that is, the discretized frequency $\omega _m$ corresponds to the effective frequency $\hat{\omega }$: $\omega _m = \hat{\omega }\Delta t$, and $\bar{z}$ denotes the complex conjugate of a complex number *z*. The number of data points *N* is assumed to be even. If this number is odd, the term *N*/2 should be replaced with $(N-1)/2$. This result (Eq. 9) illustrates the effect of the HT method on the phase-modulation in the frequency domain. Equation (9) shows that the phase reconstructed by the conventional HT method is inconsistent with the true phase for phase-modulated signals. This is because the Fourier coefficients reconstructed via the HT method $c^{\textrm{H}}_n$ are not equal to those of the true phase-modulation $c_n$. In addition, the result (Eq. 9) implies that the HT method acts as a low-pass-like filter to the phase-modulation *u*(*t*).

Here we consider two types of signals to illustrate the formula (Eq. 9) that describes the effect of Hilbert transform on the power spectra. First, let us consider the phase-modulated signal with a single frequency component $j (<N/2)$:

10 $$\begin{aligned} c_n = {\left\{ \begin{array}{ll} \alpha &{} \textrm{for} \quad n= j,\\ \bar{\alpha } &{} \textrm{for} \quad n= N-j, \\ 0 &{} \textrm{otherwise}, \end{array}\right. } \end{aligned}$$

where $\alpha$ is a non-zero complex value. If the phase-modulation frequency is lower than the effective frequency: $\omega _j < \omega _m$, the HT method perfectly reconstructs the true phase, i.e., $c^H_n= c_n$ for all *n*. Conversely, when the phase-modulation frequency is higher than the effective frequency: $j> m$, the amplitude of the reconstructed phase-modulation is half of the true phase-modulation, i.e., $c^H_n= c_n/2$ for all *n*. Indeed, Fig. 1d shows that the Fourier coefficient of the reconstructed phase-modulation $c^{\textrm{H}}_n$ is smaller than the true modulation $c_n$ near the dominant Fourier modes ($\sqrt{2} \hat{\omega }$ and $\sqrt{3} \hat{\omega }$).

**Figure 2:**
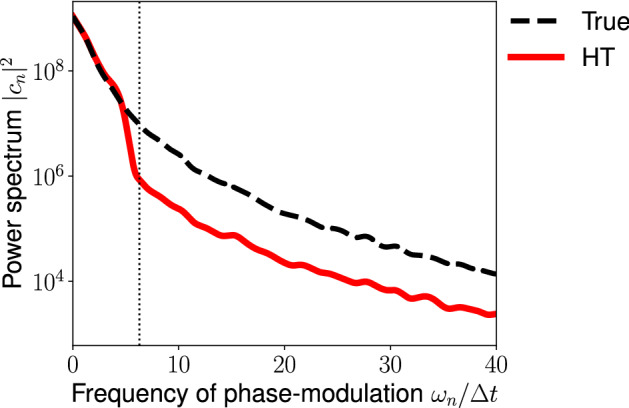
Reconstruction of the power spectrum of the phase-modulation by using the HT method. We consider the OU type phase-modulation (Eq. 11) with the parameters $k=2.0$ and $\sigma = 0.1$. The power spectrum of the phase-modulation (dashed line) was compared with that of the reconstructed phase-modularion (red). Dotted vertical line represents the effective frequency $\hat{\omega }$. The power spectrum is calculated by using the Hanning window $w[k] =0.5 - 0.5 \cos \left( 2\pi (k-0.5) / L \right) , \quad (k=1,2,\dots ,L)$, where $L=2\sqrt{N}$ is the window width and *N* is the number of data points.

In the second example, we consider the phase-modulation given by the Ornstein–Uhlenbeck process

11 $$\begin{aligned} \frac{{du (t)}}{dt} = -k u(t) + \sigma \eta (t), \end{aligned}$$

where *k* and $\sigma$ are constant and $\eta (t)$ is the Gaussian white noise with zero mean and unit variance. Figure 2 compares the power spectrum of true phase-modulation with that of the reconstructed phase-modulation by using the HT method. While the HT method accurately reconstructs the power spectrum for frequencies lower than the effective frequency, it underestimates the power spectrum for frequencies higher than the effective frequency. The coefficient (1/2) in Eq. (9) implies that the HT method underestimates the power spectrum.

We can extend the HT method to accommodate phase-modulated signals. In the following, we describe the proposed method, which consists of five steps (Algorithm 1). First, we calculate the initial guess of the phase $\phi ^{\textrm{H}}[k]$ by using the conventional HT method (Eq. 3). The Gibbs phenomenon dramatically impairs the phase reconstruction of the HT method when there is a large discrepancy between the values of the first and last point^39^. To mitigate this phenomenon, we extract the peaks from the signal and restrict the analysis to be from the first peak to the last one before applying the HT method. Second, we estimate the effective frequency $\hat{\omega }$ from the initial guess: $\hat{\omega }= (\phi ^{\textrm{H}}[N-1]-\phi ^{\textrm{H}}[0])/T$, where $T= { (N-1) \Delta t }$ is the observation duration. Third, we calculate the discrete Fourier transform of the initial guess $\{ c^{\textrm{H}}_n\}$ ($n= 1, 2, \dots , N$). Fourth, we correct the Fourier coefficient by inverting Eq. (9),

12 $$ c_{n}^{{\text{P}}}  = \left\{ {\begin{array}{*{20}l}    {2c_{n}^{{\text{H}}} } \hfill & {{\text{for}}\quad N/2 - 2m + 1 \le n \le N/2,} \hfill  \\    {2c_{n}^{{\text{H}}}  - \frac{1}{2}\bar{c}_{{n + 2m}}^{{\text{P}}} } \hfill & {{\text{for}}\quad m + 1 \le n \le N/2 - 2m,} \hfill  \\    {\text{Re} \left\{ {2c_{n}^{{\text{H}}}  + c_{{3m}}^{{\text{P}}} } \right\} + i\text{Im} \left\{ {c_{n}^{{\text{H}}}  + \frac{1}{2}c_{{3m}}^{{\text{P}}} } \right\}} \hfill & {{\text{for}}\quad n = m,} \hfill  \\    {c_{n}^{{\text{H}}}  + \frac{1}{2}\bar{c}_{{2m - n}}^{{\text{P}}}  + \frac{1}{2}c_{{n + 2m}}^{{\text{P}}} } \hfill & {{\text{for}}\quad 0 \le n \le m - 1,} \hfill  \\   \end{array} } \right. $$

where $c^{\textrm{P}}_n$ is the corrected Fourier coefficient. The remaining coefficients $c^{\textrm{P}}_n$ ($N/2 < n \le N-1$) are calculated by using the formula $c^{\textrm{P}}_{N-n}= \bar{c}^{\textrm{P}}_n$ that reflects the fact that the phase-modulation *u*[*k*] is the real signal. In the fifth step, we reconstruct the phase-modulation by calculating the inverse Fourier transform of $\{ c^{\textrm{P}}_n \}$. Then, we smooth the phase signal to remove artificial spikes in the signal. We identify outliers in the reconstructed phase using Median Absolute Deviation criteria^43^ and replace the outliers with a linear interpolation of the nearest neighbors.
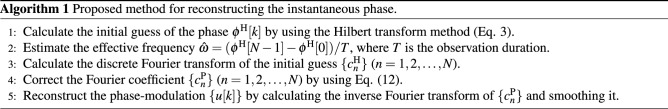


Finally, we compare the computational complexities of the conventional HT method and the proposed method for reconstructing the phase of a signal. Computational complexity, that is, the dependency of the computational time on the data size, is critical when analyzing a long signal. Let us denote the number of data points of the signal as *N*. The computational complexity of the HT method is $O(N\log N)$, because we calculate the discrete Hilbert Transform (HT) by using the discrete Fourier transform (see “Method”). Next, we evaluate the computational complexity of the proposed method. First, the proposed method computes the HT: $O(N\log N)$ (Step 1 in Algorithm 1). Next, the effective frequency is calculated: *O*(1) (Step 2). Then, the discrete Fourier transform is computed: $O(N\log N)$ (Step 3) and the coefficients of the Fourier transform are corrected: *O*(*N*) (Step 4). Finally, the method reconstructs the phase-modulation by calculating the inverse Fourier transform: $O(N\log N)$ and smoothing it: $O(N\log N)$ (Step 5). Therefore, the computational complexity of the proposed algorithm is $O(N\log N)$, which is comparable to that of the conventional method. In terms of the complexity, the proposed algorithm is better than the iterative Hilbert transform embedding (IHTE)^33^: $O(N^2)$, which is one of the state-of-the-art methods for reconstructing the phase.

### Reconstruction performance of the proposed method

Here, we examine whether the proposed method can accurately reconstruct the instantaneous phase from an observed signal. First, we consider an oscillatory signal (Eq. 6) with a constant amplitude $A_0 = 1$ The sampling time interval and the duration of the simulation are $\Delta t =0.01$ and $T=200$, respectively, unless otherwise stated.

We evaluated the performance of the phase reconstruction by analyzing the synthetic data based on two types of phase-modulated signals. The first signal is a quasi-periodic phase-modulation,

13 $$\begin{aligned} u(t) = b \left( \sin \sqrt{2} \hat{\omega } t + \cos \sqrt{3} \hat{\omega } t \right) , \end{aligned}$$

where *b* is the amplitude of the phase-modulation. The second signal is the Ornstein–Uhlenbeck (OU) type phase-modulation (Eq. 11).

We applied the proposed method to a signal with quasi-periodic phase-modulation (Fig. 1a). Figure1c compares the phase reconstructed via the proposed method (blue) with that reconstructed via the conventional HT method (red). While the proposed method accurately reconstructs the phase-modulation, the conventional method cannot reconstruct it. In addition, we compared the power spectrum of the phase-modulation with that of the reconstructed phase-modulations (Fig. 1d). We found that the proposed method can reconstruct a phase-modulation whose power spectrum is consistent with the true power spectrum. However, a small peak (frequency $\sim$ 9.2) appears in the power spectrum of the proposed method (Fig. 1d). This error might be due to the nonlinear effect $O(\epsilon ^2)$ ignored in the derivation of the proposed method.

**Figure 3:**
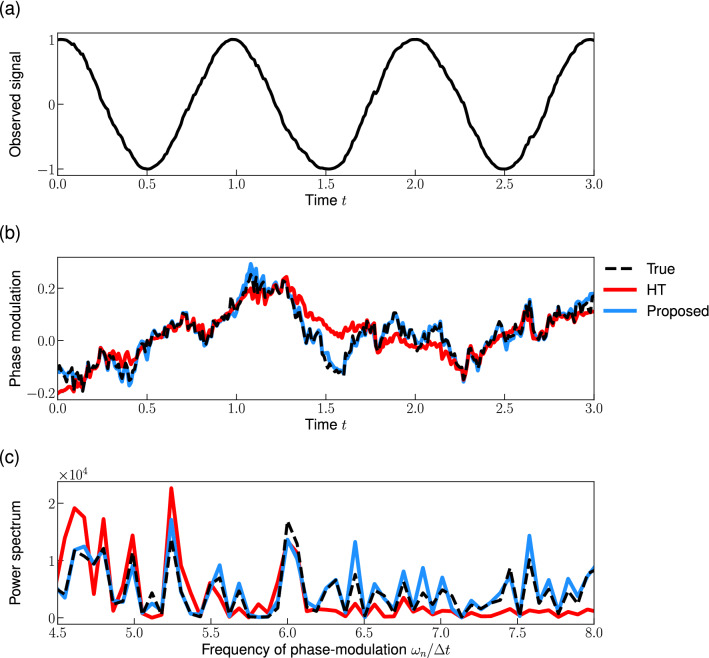
Reconstructing the instantaneous phase by the proposed method: a signal with the OU type phase-modulation. (**a**) Observed signal *x*(*t*) given by Eqs. (6) and (11) with $A_0 = 1$. (**b**) Phase-modulation *u*(*t*). (**c**) Power spectrum of the phase-modulation. Dashed lines represent the true phase-modulation *u*(*t*) in (**b**) and its power spectrum in (**c**). The red and blue lines represent the reconstructions by the conventional HT method and the proposed method, respectively. Parameters are $k=2.0$, and $\sigma ^2= 0.28$. Note that we plotted a part of signal and the start time of the plot is redefined as 0.

Next, we applied the proposed method to a signal with the OU type phase-modulation (Fig. 3a). Similar to the case of the quasi-periodic modulation, the proposed method can reconstruct the phase-modulation (Fig. 3b) and its power spectrum (Fig. 3c) accurately. While the conventional HT method can track the slow trend of the phase fluctuation, it cannot accurately reconstruct the phase-modulation.

**Figure 4:**
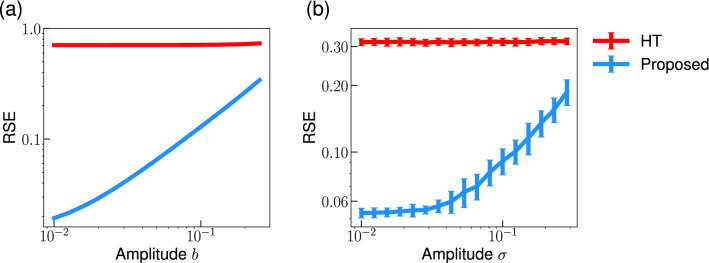
Effect of the amplitude of phase-modulation on the phase reconstruction error. (**a**) Quasi-periodic phase-modulation (Eq. 13). (**b**) OU type phase-modulation (Eq. 11). We plotted the mean and standard deviation of the errors calculated from 100 trials in (**b**). Parameters are set as $\hat{\omega }= 2\pi$ and $k=2.0$ in (**b**).

Furthermore, we examined whether the proposed method can reconstruct the phase given a larger phase-modulation. We quantified the phase reconstruction performance based on the relative squared error (RSE):

14 $$\begin{aligned} \textrm{RSE} = \frac{ \sum _{k=1}^N (\hat{u}[k]- u[k])^2}{ \sum _{k=1}^N u^2[k]}, \end{aligned}$$

where $\hat{u}[k]$ (*u*[*k*]) represents the reconstructed (true) phase-modulation, and *N* is the number of data points. Figure 4a shows that the proposed method consistently performs better than the conventional HT method for a signal with a quasi-periodic phase-modulation for a range of the phase-modulation amplitude *b*. Nevertheless, the error of the proposed method increases with increasing phase-modulation amplitude. The performance deterioration may be due to the nonlinear effects, i.e., $O\left( \varepsilon ^2\right)$, which we neglected in the derivation of the method. Similarly, the proposed method consistently performs better than the HT method for a signal with an OU type phase-modulation even when the phase-modulation is not small (Fig. 4b). Even though we assumed a small phase-modulation to derive the proposed method, the results (Fig. 4) suggest that the proposed method provides better performance than the HT method for signals with moderate phase-modulations.

Then, we examined whether the proposed method can reconstruct the phase from a signal with the amplitude and phase-modulations. We consider a signal whose amplitude is modulated by a sum of sinusoidal functions

15 $$\begin{aligned} x(t)= A(t) \cos \left( \hat{\omega } t + u(t) \right) , \end{aligned}$$

where $\hat{\omega }$ is the effective frequency,

16 $$\begin{aligned} A(t) = 1+ 0.2\left( \cos 0.6 \hat{\omega } t + \sin 0.7 \hat{\omega } t \right) , \quad u(t)= 0.2 \left( \sin \sqrt{2} \hat{\omega } t+ \cos \sqrt{3} \hat{\omega } t \right) . \end{aligned}$$

**Figure 5:**
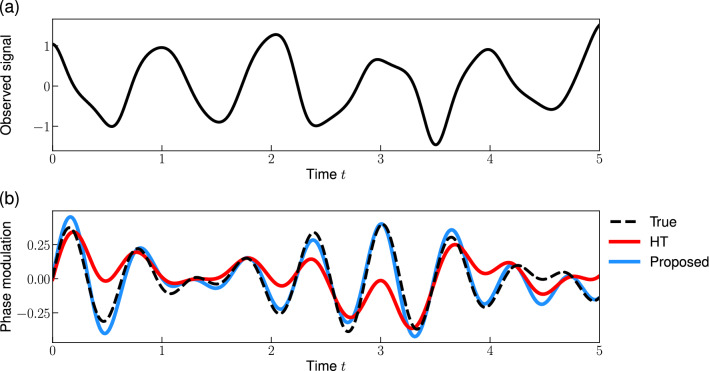
Reconstructing the instantaneous phase by the proposed method: a signal with amplitude and phase-modulation. (**a**) Observed signal *x*(*t*) (Eq. 15). (**b**) Phase-modulation *u*(*t*). The dashed line represents the true phase-modulation *u*(*t*), and the red and blue line represents its reconstruction by the HT method and the proposed method, respectively. Note that we plotted a part of signal and the start time of the plot is redefined as 0.

Figure 5 demonstrates that while the proposed method can accurately reconstruct the instantaneous phase from the amplitude and phase-modulated signal, the conventional HT method cannot. This result can be understood as follows. Bedrosian’s theorem^42^ states that the HT of the product of a high-pass and a low-pass signal with non-overlapping spectra is equal to the product of the low-pass signal and the HT of the high-pass signal (see Eq. 27). This theorem implies that the slow (or low-pass filtered) amplitude modulation (Eq. 5) will not impair the phase reconstruction by the conventional HT method. Thus, it is natural to expect that the proposed method works even when we observe the weakly phase-modulated signals.

Furthermore, we examined whether the proposed method is robust against slow amplitude modulation. We consider an amplitude and phase-modulated signal (15) with

17 $$\begin{aligned} A(t) = 1+ r \cos \nu t, \quad u(t)= 0.2 \left( \sin \sqrt{2} \hat{\omega } t + \cos \sqrt{3} \hat{\omega } t \right) . \end{aligned}$$

**Figure 6:**
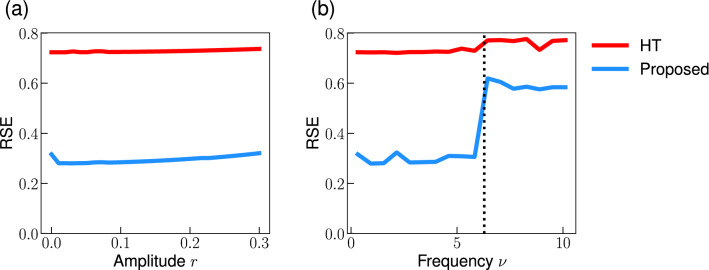
Effect of the amplitude modulation on the phase reconstruction error. Dependence of the error on the amplitude *r* (**a**) and the frequency $\nu$ (**b**) of the amplitude modulation. Parameter were set as $\nu = 4.4$ in (**a**) and $r= 0.1$ in (**b**). The dotted vertical line in (**b**) represents the effective frequency $\hat{\omega }= 2 \pi$.

Figure 6 shows the dependence of the reconstruction error on the amplitude *r* and frequency $\nu$ of the amplitude modulation. The error does not depend on the amplitude, which indicates that the proposed method works even for signals with moderate amplitude modulation (Fig. 6a). While the error does not depend on the frequency $\nu$ in the range of $\nu < \hat{\omega }$ (Fig. 6b), it increases when the frequency becomes larger than the effective frequency $\hat{\omega }$. Nevertheless, the error of the proposed method is smaller than the HT method. Overall, these results suggest that the proposed method improves the phase reconstruction even for the signals with amplitude modulation.

**Figure 7:**
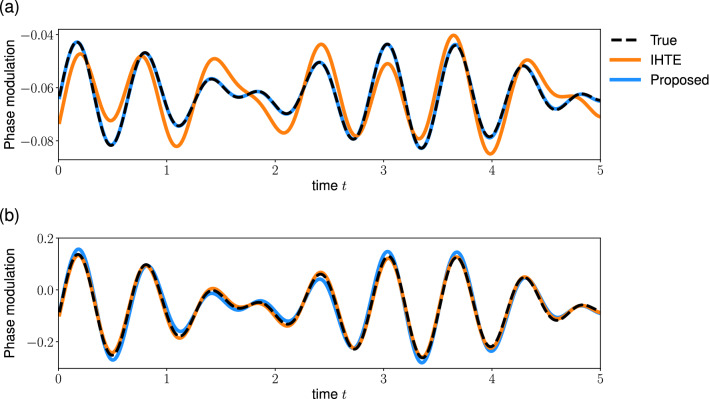
Comparison of the phase reconstruction methods: the signals with quasi-periodic phase-modulation. The proposed method and the IHTE method were applied to the phase-modulated signal (Eq. 6) with (**a**) small or (**b**) large quasi-periodic modulation (Eq. 13). The dashed line represents the true phase-modulation *u*(*t*), and the blue and orange lines represent its reconstruction by the proposed and the IHTE method, respectively. Parameters are $b=0.02$ in (**a**), $b=0.1$ in (**b**), and $A_0= 1$ and $\hat{\omega }=2\pi$ in both panels. Note that we plotted a part of signal and the start time of the plot is redefined as 0.

**Figure 8:**
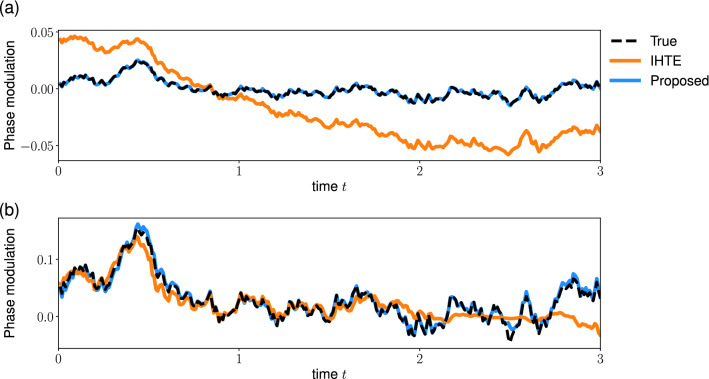
Comparison of the phase reconstruction methods: the signals with OU type phase-modulation. The proposed method and the IHTE method were applied to the phase-modulated signal (Eq. 6) with (**a**) small or (**b**) large OU type phase-modulation (Eq. 11). The dashed line represents the true phase-modulation *u*(*t*), and the blue and orange line represent its reconstruction by the proposed and the IHTE method, respectively. Parameters are $\sigma =0.02$ in (**a**), $\sigma =0.2$ in (**b**), and $A_0= 1$, $\hat{\omega }=1$, and $k=2$ in both panels. Note that we plotted a part of signal and the start time of the plot is redefined as 0.

Finally, we compare the phase reconstruction by the proposed method with that by the iterative Hilbert transform embedding (IHTE) method^33^. For the IHTE method, we used the proxi-phase based on the analytic signal (Eq. 3) and fixed the number of iteration *K* as 20. We stopped the iteration if the IHTE method returns an error. Here, we analyzed the phase-modulated signals (Eq. 6) with a constant amplitude: $A_0= 1$. Figure 7 demonstrates the phase reconstructions by the proposed method (blue) and the IHTE method (orange) from the quasi-periodic phase-modulated signals (Eq. 13). When the phase-modulation is small (Fig. 7a), the proposed method can reconstruct the phase more accurately than the IHTE method. The relative squared error (Eq. 14) is 0.007 and 0.06 for the proposed method and the IHTE method, respectively. In contrast, when the phase-modulation is large (Fig. 7b), the IHTE method performs better than the proposed method. The error is 0.1 and 0.03 for the proposed method and the IHTE method, respectively. Next, we analyzed the noisy (OU type) phase-modulated signals (Eq. 11). When the noise amplitude of the phase-modulation is much smaller than the effective frequency $\hat{\omega }$, the result is similar to the quasi-periodic phase-modulated signal (Fig. 7). The proposed method performs better for a signal with small phase-modulation, whereas the IHTE method performs better for a signal with large phase-modulation (data not shown). In contrast, the proposed method reconstructs the phase more accurately than the IHTE for noisy phase-modulated signals (Fig. 8). The relative squared error of the proposed and the IHTE method was 0.07 and 2 for a signal with small phase-modulation (Fig. 8a), and 0.1 and 0.7 for a signal with large phase-modulation (Fig. 8b), respectively. These results suggest that the proposed method is suitable for the signals with small phase-modulation or noisy phase-modulation compared to the IHTE method. Note that a large error does not necessarily mean the poor performance of the IHTE method due to the difference in the phase definition (see “Discussion”).

### Detecting a phase shift from an observed signal

Biological oscillatory systems often exhibit “phase shifts”, that is, a rapid change in the phase of a rhythm. For example, the phase of a circadian rhythm can change as a result of light exposure^44^. It would be useful to develop a method for detecting the phase shifts in oscillatory signals. We examined whether the proposed method is potentially useful for detecting the phase shifts in data. As a minimal model, we consider a single oscillator exhibiting a phase shift:

18 $$\begin{aligned} x(t) = \cos \left( \hat{\omega }_1 t + u(t) \right) , \quad \frac{du(t)}{dt} = {\left\{ \begin{array}{ll} \sigma \eta (t) &{} \textrm{for} \quad t \notin [T_c, T_c+\Delta _T], \\ \left( \hat{\omega }_2- \hat{\omega }_1 \right) + \sigma \eta (t) &{} \textrm{for} \quad t \in [T_c, T_c+\Delta _T], \end{array}\right. } \end{aligned}$$

where the interval $[T_c, T_c+\Delta _T]$ represents the change period, i.e., the period in which the phase of the oscillator shifts with a frequency $\hat{\omega }_2$, and $\eta (t)$ is the Gaussian white noise with zero mean and unit variance. The synthetic data were simulated with a total duration $T= 10$ and a change time $T_c= 5.0$.

**Figure 9:**
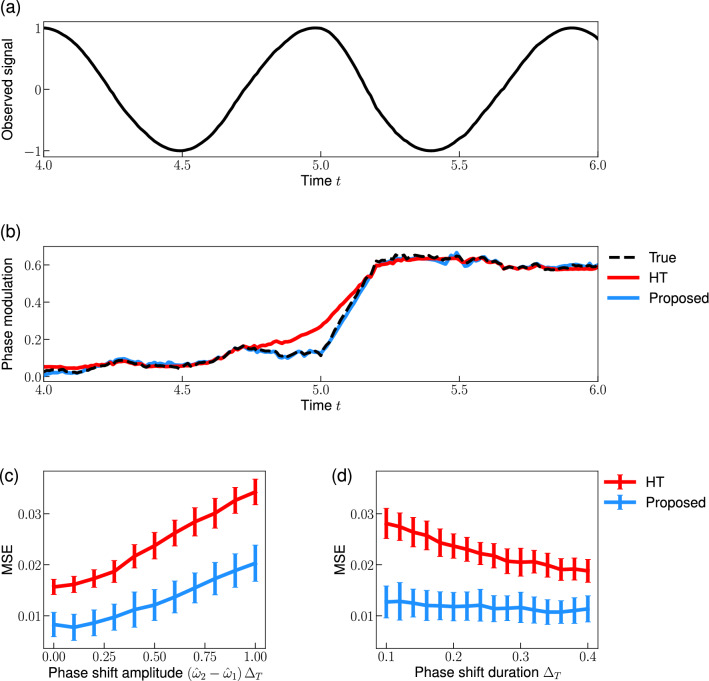
Detecting the phase shift from an oscillatory signal. (**a**) Observed signal *x*(*t*) (Eq. 18). (**b**) Phase-modulation *u*(*t*). (**c**,**d**) Dependence of the phase reconstruction error on the phase shift amplitude in (**c**) and the phase shift duration in (**d**). We plotted the mean and standard deviation of the mean squared errors (MSEs) calculated from 100 trials in (**c**,**d**). Parameters were set as the effective frequency $\hat{\omega }_1= 2\pi$, the noise variance $\sigma = 0.1$, the shift amplitude $(\hat{\omega }_2- \hat{\omega }_1) \Delta _T= 0.5$ in (**a**,**b**,**d**), and the the shift duration $\Delta _T = 0.2$ in (**a**–**c**).

Figure 9a shows an observed signal before and after the change period, with the frequency increasing after $t= 5.0$. The proposed method can accurately track the change in the phase-modulation induced by the phase shift (Fig. 9b: blue). Conversely, it is difficult for the conventional HT method to infer the change period because of the smoothing effect in the reconstructed phase (Fig. 9b: red). Furthermore, we compared the phase reconstruction error of the proposed method with that of the conventional HT method. We calculate the mean squared error between the reconstructed phase and the true phase. The proposed method achieved a smaller error than the HT method across a range of phase shift amplitude $(\hat{\omega }_2- \hat{\omega }_1) \Delta _T$ (Fig. 9c). Similar to the previous result concerning the reconstruction error (Fig. 4), the errors of these methods increase with increasing phase shift amplitude. In addition, we examined the dependency of the error on the duration of the phase shift $\Delta _T$ when the phase shift amplitude is fixed. The error of the HT method increases as the shift duration decreases (Fig. 9d: red). Conversely, the error of the proposed method is small even for a signal with a small duration $\Delta _T$ (Fig. 9d: blue). This result suggests that the proposed method is more suitable for detecting rapid phase shifts than the HT method.

## Discussion

We proposed an extension of the Hilbert Transform (HT) method for reconstructing the phase from an observed signal. We addressed a limitation of the conventional HT method, that is, the conventional method has been proven to work only for the narrow band signals. We demonstrated that the conventional HT method cannot accurately reconstruct the interpretable phase from phase-modulated signals. Conversely, our method can extract the phase from these types of signals (Figs. 1, 3, 4). Furthermore, we have demonstrated the performance of the proposed method by using the simulated data with the amplitude- and phase-modulated signals (Figs. 5, 6). Consequently, the extended HT method is a promising tool for investigating synchronization phenomena through analyzing oscillatory signals in biological systems.

A recent method for reconstructing the phase from a signal is the iterative Hilbert transform embedding (IHTE)^33,34,45^. While both the IHTE method and the proposed method aim to improve the HT method, there are several important differences between the two methods. First, the definition of phase is different between these methods. The proposed method assumes that the signal *x*(*t*) is decomposed into the amplitude *A*(*t*) and phase $\phi (t)$ by using the cosine function: $x(t)= A(t) \cos (\phi (t))$. This definition can capture the non-stationarity in the signal and is used in the signal processing literature^37,40,41^. In contrast, the IHTE method assumes that the signal is described as follows: $x(t)= S(\phi (t))$, where $S(\phi )$ is the waveform function. This formulation allows us to incorporate the complex waveform, whereas it cannot handle the non-stationarity. Second, we compared the reconstruction performance of the proposed method and the IHTE method. The result suggests that the proposed method is suitable for the signals with noisy phase-modulation (Fig. 8), whereas the IHTE method achieved better performance for the signals with large quasi-periodic phase-modulation (Fig. 7b). Note that the deviation from the true phase might not indicate the poor performance of the IHTE method due to the difference in the phase definition. In addition, Gengel and Pikovsky developed the IHTE method for noisy signals^45^. A more systematic evaluation of these methods is beyond the scope of this study. Finally, the proposed method is suitable for analyzing long signals. The computational complexity of the proposed method is comparable to the original HT method: $O(N \log N)$, where *N* is the number of data points. By contrast, the complexity of the IHTE method is $O(K N^2)$, where $K \sim 20$ is the number of iterations of the IHTE.

There are limitations to the proposed method, offering the opportunity for future improvements. First, we assumed that the phase-modulation *u*(*t*) is so small that higher-order terms are negligible. In addition, we assumed that the amplitude of the signal is approximately constant. Despite these assumptions, the numerical results (Figs. 4, 5, 6) indicate that the proposed method is, to some extent, robust against violations of these assumptions. As shown in Fig. 6, the amplitude modulation of higher frequency can impair the performance of the proposed method. It is difficult to derive an approximate expression of the phase obtained by the HT method, e.g., Eq. (29), when the amplitude varies in time. Further studies are required to develop a phase reconstruction method that is robust to amplitude modulations. For example, it would be interesting to extend the proposed method by using the state-space model^46–49^ to capture the amplitude modulation in a signal. Lastly, our method assumed a specific form of the observation signals, $x(t)= A(t) \cos (\hat{\omega } t+ u(t) )$. While this assumption seems to be reasonable for rodent local field potential (LFP) and human electroencephalogram (EEG) recordings^49^, it might fail for the signals from a nonlinear oscillator^21^ or ECG measurement^24,50^. The spectral domain of the amplitude *A*(*t*) and phase-modulation *u*(*t*) would be highly overlapping for these signals, which impairs the accuracy of the proposed method. Thus, the proposed method should be extended in future studies to analyze the signals whose waveform deviates from the cosine function.

Another future research direction is to apply the proposed method to a real-world dataset. We have shown that the proposed method can accurately reconstruct the high-frequency component compared with the conventional HT method. Therefore, the proposed method should be able to improve the estimation performances of the phase equations from the observed signals in biological systems. Moreover, the proposed method can identify the phase shifts in oscillatory signals more accurately than the conventional method (Fig. 9). It would be an interesting future study to identify the phase shifts in the circadian rhythms using the proposed method.

## Method

### Discrete Hilbert transform

We used the Hilbert Transform (HT) for discrete signals, i.e., we used the discrete HT to analyze the signals. In this subsection, we describe the definition and properties of the discrete HT. Let $X(\omega )$ be the Fourier transform of a continuous signal *x*(*t*):

19 $$\begin{aligned} x(t) = \int _{-\infty }^{\infty } d\omega X(\omega ) e^{i\omega t}. \end{aligned}$$

The HT of *x*(*t*) (Eq. 2) can be written by using its Fourier transform^36^

20 $$\begin{aligned} H\left[ {x(t)}\right] = \int _{-\infty }^{\infty } d\omega \left( -i \cdot \textrm{sgn}(\omega )\right) X(\omega ) e^{i\omega t}, \end{aligned}$$

where $\textrm{sgn}$ denotes the sign function defined as

21 $$\begin{aligned} \textrm{sgn}(x) = {\left\{ \begin{array}{ll} -1 &{} \textrm{for} \quad x<0, \\ 0 &{} \textrm{for} \quad x=0, \\ 1 &{} \textrm{for} \quad x>0. \end{array}\right. } \end{aligned}$$

The discrete HT is defined to satisfy the property similar to Eq. (20). We consider the inverse discrete-time Fourier transform of a signal (sequence) $x[k]=x(k\Delta t)$:

22 $$\begin{aligned} x[k] = \sum _{n=0}^{N-1} X_n e^{ik\omega _n}, \end{aligned}$$

where $X_n$ is the discrete Fourier transform of *x*[*k*], and $\omega _n= 2n \pi /N$ is the *n*-th frequency. The discrete HT of *x*[*k*] is defined as

23 $$\begin{aligned} H_d\left( {x[k]}\right) = \sum _{n=0}^{N-1} (-i S_n) X_n e^{ik\omega _n}, \end{aligned}$$

where $$S_n$$ is

24 $$\begin{aligned} S_n = {\left\{ \begin{array}{ll} 0 &{} \textrm{for } \quad n=0,\\ 1 &{} \textrm{for } \quad 0< n \le N/2, \\ -1 &{} \textrm{for } \quad N/2 < n \le N-1. \end{array}\right. } \end{aligned}$$

Again, we assume that the number of data points *N* is even. If this number is odd, the term *N*/2 should be replaced with $(N-1)/2$. From this definition (Eqs. 23, 24), we can derive the formula implying that the discrete HT can also reconstruct the phase of the sinusoidal wave,

25 $$\begin{aligned} H_d\left( { \cos (\omega k\Delta t ) }\right) = \sin (\omega k\Delta t ), \qquad H_d\left( { \sin (\omega k\Delta t ) }\right) = -\cos (\omega k\Delta t ), \end{aligned}$$

where $0< \omega < \pi$ is the frequency parameter.

Next, we introduce Bedrosian’s theorem, which states that the HT of the product of a high-pass signal and a low-pass signal with non-overlapping spectra is given by the product of the low-pass signal and the HT of the high-pass signal. Formally, it is written as follows:

#### Theorem

(Bedrosian’s theorem for the discrete Hilbert transform^36,51^) *Let* *x*[*k*] *and* *y*[*k*] ($k=0,1,\dots , N-1$) *be sequences with their Fourier transform* $X_n$ *and* $Y_n$ ($n=0,1,\dots , N-1$), *respectively. If there exists an integer* $0< m < N/2$ *such that*

26 $$\begin{aligned} \begin{array}{cl} X_n = 0 &{}\qquad \textrm{for} \quad m \le n \le N-m, \\ Y_n = 0 &{}\qquad \textrm{for} \quad 0 \le n \le m-1,\ \ N-m+1 \le n \le N-1, \end{array} \end{aligned}$$

*then the discrete Hilbert transform of the product of* *x*[*k*] *and* *y*[*k*] *is written as*

27 $$\begin{aligned} H_d\left( {x[k] y[k]}\right) = x[k] H_d\left( {y[k]}\right) . \end{aligned}$$

### Analysis of phase-modulation reconstructed via the conventional Hilbert transform method

Here, we analyze the phase-modulation reconstructed via the conventional HT method. The aim of this subsection is to derive a formula (Eq. 9) that characterizes the relationship between the phase-modulation and its reconstruction. Let us assume that we observe a weakly phase-modulated signal

28 $$\begin{aligned} x[k] = \cos \phi [k], \end{aligned}$$

where $\phi [k]= \hat{\omega } k\Delta t + u[k]$ is the instantaneous phase at time $k\Delta t$, $\hat{\omega }$ is the effective frequency, and *u*[*k*] is a small phase-modulation: $\varepsilon =\max _k \left| u[k]\right| \ll 1$. We shifted the time origin to satisfy $\phi [0]= 0$ in the analysis. Note that the amplitude can be set as 1 ($A_0= 1$) without loss of generality.

The HT method reconstructs the phase via the argument of the analytic signal (Eq. 3). Substituting Eq. (28) into Eq. (3), we obtain

29 $$\begin{aligned} \phi ^{\textrm{H}}[k]= & {} \arg \left[ \ \cos \phi [k] + i H_d\left( { \cos \phi [k] }\right) \ \right] = \arg \left[ \ \cos \left( \hat{\omega } k\Delta t+ u[k] \right) + i H_d\left( { \cos \left( \hat{\omega } k\Delta t + u[k] \right) }\right) \ \right] \nonumber \\\approx & {} \arg \left[ \ \cos ( \hat{\omega } k\Delta t ) - u[k] \sin ( \hat{\omega } k\Delta t ) + i H_d\left( { \cos ( \hat{\omega } k\Delta t ) - u[k] \sin ( \hat{\omega } k \Delta t ) }\right) \ \right] \nonumber \\= & {} \arg \left[ \left\{ \cos (\hat{\omega } k\Delta t)+ i \sin (\hat{\omega } k\Delta t) \right\} - \left\{ u[k] \sin (\hat{\omega } k\Delta t) + i H_d\left( u[k] \sin (\hat{\omega } k\Delta t) \right) \right\} \right] \nonumber \\= & {} \hat{\omega } k\Delta t + \arg \left[ 1 - e^{-i \hat{\omega } k \Delta t} \left\{ u[k] \sin ( \hat{\omega } k \Delta t) + iH_d\left( {u[k] \sin ( \hat{\omega } k \Delta t) }\right) \right\} \right] \nonumber \\\approx & {} {\hat{\omega }} k\Delta t - \textrm{Im} \left[ e^{-i{\hat{\omega} }k\Delta t} \left\{ u[k] \sin ( {\hat{\omega} }k\Delta t )+ i H_d\left( {u[k] \sin ( {\hat{\omega }}k\Delta t) }\right) \right\} \right] , \end{aligned}$$

where the approximation symbol $\approx$ represents that the higher order terms $O(\epsilon ^2)$ are neglected, and $\textrm{Im}[z]$ denotes the imaginary part of a complex number *z*. Hence, the phase-modulation reconstructed via the conventional HT method $u^{\textrm{H}}[k]:=\phi ^{\textrm{H}}[k] - \hat{\omega }k\Delta t$ can be written as follows:

30 $$\begin{aligned} u^{\textrm{H}}[k] \approx f\left( u[k] \right) := -\textrm{Im} \left[ e^{-i{\hat{\omega }}k\Delta t} \left\{ u[k] \sin ( {\hat{\omega }}k\Delta t )+ i H_d\left( {u[k] \sin ( \hat{\omega }k\Delta t) }\right) \right\} \right] . \end{aligned}$$

To analyze the discrete HT in Eq. (30), we consider the discrete Fourier series of the phase-modulation

31 $$\begin{aligned} u[k] = \sum _{n= 0}^{N-1} c_n e^{i k \omega _n} = \sum _{n=0}^{N/2} v_n[k], \end{aligned}$$

where $\omega _n= 2 \pi n/N$ is a frequency of the *n*-th Fourier component,

$$\begin{aligned} v_n[k] = {\left\{ \begin{array}{ll} c_n e^{ik\omega _n} &{} \textrm{for} \quad n= 0 \quad \textrm{or} \quad n= N/2, \\ c_n e^{ik\omega _n} + \bar{c}_n e^{-ik\omega _n} &{} \textrm{otherwise}, \end{array}\right. } \end{aligned}$$

is the *n*-th frequency component. For the derivation of Eq. (31), we used the formula $c_{N-n}= \bar{c}_n$, which reflects the fact that the phase-modulation is a real signal. Here, the number of data points *N* is assumed to be even. If this number is odd, the term *N*/2 should be replaced with $(N-1)/2$. Due to the linearity of the HT, the reconstructed phase-modulation can be written as

32 $$\begin{aligned} u^{\textrm{H}}[k] \approx \sum _{n=0}^{N/2} f \left( v_n[k]\right) . \end{aligned}$$

We can further calculate Eq. (32) for each term $f\left( v_n[k]\right)$ by dividing three cases based on a frequency index $m=\hat{\omega }N \Delta t / 2\pi$ that corresponds to the effective frequency $\hat{\omega }$.

**Case 1**: Low frequency modulation: $0\le n \le m-1$. Using Bedrosian’s theorem (Eq. 27) and Eq.(25), we have
  33 $$\begin{aligned} H_d\left( {v_n[k] \sin (\hat{\omega } k \Delta t)}\right) = - v_n[k]\cos ( \hat{\omega } k \Delta t ). \end{aligned}$$
  Substituting Eq. (33) into Eq. (30), we obtain
  34 $$\begin{aligned} f \left( v_n[k]\right) = v_n[k]. \end{aligned}$$
**Case 2**: Middle frequency modulation: $$ n=m$$. We have
  35 $$\begin{aligned} H_d\left( {v_n[k] \sin (\hat{\omega } k \Delta t)}\right)= & {} H_d\left( {\left( c_n e^{ik\omega _n} + \bar{c}_n e^{-ik \omega _n } \right) \frac{e^{ik\omega _n} - e^{-ik\omega _n}}{2i}}\right) \nonumber \\= & {} -\frac{1}{2} \left( c_n e^{2ik\omega _n } + \bar{c}_n e^{-2ik\omega _n }\right) , \end{aligned}$$
  where we used the definition of the discrete HT (Eqs. 23, 24). Substituting Eq. (35) into Eq. (30), we obtain
  36 $$\begin{aligned} f \left( v_n[k]\right)= & {} \textrm{Im} \left[ -e^{ik\omega _n} \left\{ \left( c_n e^{ik\omega _n} + \bar{c}_n e^{-ik\omega _n} \right) \frac{e^{ik\omega _n} - e^{-ik\omega _n}}{2i} - \frac{i}{2} \left( c_n e^{2ik\omega _n} + {\bar{c}}_n e^{-2ik\omega _n} \right) \right\} \right] \nonumber \\= & {} \textrm{Im} \left[ \frac{i}{2} \left\{ c_n e^{ik\omega _n} - c_n e^{-ik\omega _n} + {\bar{c}}_n e^{-ik\omega _n} + c_n e^{ik\omega _n} \right\} \right] \nonumber \\= & {} \left( \frac{3}{4}c_n -\frac{1}{4} {\bar{c}}_n \right) e^{i k \omega _n } - \left( \frac{1}{4}c_n -\frac{3}{4}\bar{c}_n \right) e^{ - i k \omega _n }. \end{aligned}$$
**Case 3**: : High frequency modulation: $m+1\le n \le N/2$. Using Bedrosian’s theorem (Eq. 27) and the definition of the discrete HT (Eqs. 23, 24), we have
  37 $$\begin{aligned} H_d\left( {v_n[k] \sin (\hat{\omega } k \Delta t)}\right) = {\left\{ \begin{array}{ll} i \left( - c_n e^{ik\omega _{n} } + \bar{c}_n e^{-ik\omega _{n}} \right) \sin (\hat{\omega } k \Delta t ) &{} \textrm{for} \quad m+1 \le n < N/2, \\ - i c_n e^{ik\omega _{n} } \sin (\hat{\omega } k \Delta t ) &{} \textrm{for} \quad n=N/2. \end{array}\right. } \end{aligned}$$
  Substituting Eq. (37) into Eq. (30), we obtain
  38 $$\begin{aligned} f \left( v_n[k]\right)= & {} {\left\{ \begin{array}{ll} \textrm{Im}\left[ -e^{-ik\omega _m} \left\{ c_n e^{ik\omega _n} + {\bar{c}}_n e^{-ik\omega _n} + c_n e^{ik\omega _n} - {\bar{c}}_n e^{-ik\omega _n} \right\} \sin {\hat{\omega }}k\Delta t \right] &{} \textrm{for} \quad m+1 \le n < N/2, \nonumber \\ \textrm{Im}\left[ -e^{-ik\omega _m} \left\{ c_n e^{ik\omega _n} + c_n e^{ik\omega _n} \right\} \sin {\hat{\omega }}k\Delta t \right] &{} \textrm{for} \quad n=N/2, \nonumber \end{array}\right. }\nonumber \\= & {} \textrm{Im}\left[ i c_n e^{ik\omega _n} \left( 1-e^{-2i \omega _m}\right) \right] \quad ( \textrm{for} \quad m+1 \le n \le N/2) \nonumber \\= & {} \frac{1}{2}c_n e^{ik\omega _n} + \frac{1}{2}{\bar{c}}_n e^{-ik\omega _n} - \frac{1}{2} c_n e^{i k \omega _{n - 2m}} - \frac{1}{2} {\bar{c}}_n e^{-i k \omega _{n-2m} }. \end{aligned}$$

Substituting Eqs. (34), (36), and (38) into Eq. (32), we obtain the Fourier series of the phase-modulation reconstructed by the conventional HT method

39 $$\begin{aligned} u^{\textrm{H}}[k]\approx & {} c_{0} - \frac{1}{2}\bar{c}_{2m} - \frac{1}{2}c_{2m} + \left\{ \sum _{n=1}^{m-1}\left( c_{n} - \frac{1}{2} \bar{c}_{2m-n} - \frac{1}{2} c_{n+2m} \right) e^{ik\omega _n} + \left( \frac{3}{4} c_{m} -\frac{1}{4}\bar{c}_{m} - \frac{1}{2}c_{3m} \right) e^{ik \omega _m } \right. \nonumber \\{} & {} + \left. \sum _{ n= m+1 }^{N/2-2m}\left( \frac{1}{2}c_{n} - \frac{1}{2}c_{n+2m}\right) e^{ik\omega _n} + \sum _{n=N/2-2m +1}^{N/2}\frac{1}{2}c_{n}e^{ik\omega _n} + \mathrm{c.c.} \right\} , \end{aligned}$$

where c.c denotes the complex conjugate of the terms in the curly brackets. In the derivation of Eq. (39), we assumed that the sampling interval is small enough, i.e., $N \ge 6m+2$. Finally, we obtain Eq. (9) by rearranging Eq. (39). Note that we can also obtain a similar formula between $c_n$ and $c^H_n$ (Eq. 9) even when the amplitude *A*(*t*) changes slowly, i.e., the amplitude is a low-pass signal whose spectrum does not overlap with the spectra of $\cos \phi (t)$.

### Phase reconstruction based on the discrete Hilbert transform

Here, we describe the conventional HT method for reconstructing the phase $\phi ^{\textrm{H}}[k]$ from a discrete oscillatory signal *x*[*k*]. First, we pre-process the signal in order to mitigate the Gibbs phenomenon: we detect the first and the last peak points of the signal, and delete all of the data points prior to the first peak or after the last peak. Next, we calculate the Fourier transform of *x*[*k*] to obtain $X_n$. Then, we calculate the discrete HT, $H_d\left( {x[k]}\right)$, according to Eq. (23). Finally, we reconstruct the phase by calculating the argument of the analytic signal $\phi ^{\textrm{H}}[k] := \arg \left( x[k] + iH_d\left( {x[k]}\right) \right)$.

## Data Availability

The datasets and simulation codes for generating the data are available at https://github.com/AkariMatsuki/AnExtendedHilbertTransform.git.

## Code availability

The code of the extended Hilbert transform is available at https://github.com/AkariMatsuki/AnExtendedHilbertTransform.git.
